# Supplementary material for: Comparative genomics provides new insights into the diversity, physiology, and sexuality of the only industrially exploited tremellomycete: Phaffia rhodozyma
Source: BMC Genomics. 2016 Nov 9;17:901. doi: 10.1186/s12864-016-3244-7 (PMC5103461; doi:10.1186/s12864-016-3244-7)
Supplement: Additional file 6: — List of orphan genes with links to PFAM (related to Additional file 1: Table S1). (ZIP 1428 kb) [file 12864_2016_3244_MOESM6_ESM.zip › PFAM_UNIQUE/G02171_P.html]

#                                                                            --- full sequence --- -------------- this domain -------------   hmm coord   ali coord   env coord
  
# target name        accession   tlen query name           accession   qlen   E-value  score  bias   #  of  c-Evalue  i-Evalue  score  bias  from    to  from    to  from    to  acc description of target
  
#------------------- ---------- ----- -------------------- ---------- ----- --------- ------ ----- --- --- --------- --------- ------ ----- ----- ----- ----- ----- ----- ----- ---- ---------------------
  
G02171\_P             -            314 Competence           PF03772.11   271       2.6    5.7  14.6   1   2    0.0028       2.4    5.8   1.8   196   265     9    78     2    83 0.83 - Competence protein  
G02171\_P             -            314 Competence           PF03772.11   271       2.6    5.7  14.6   2   2    0.0041       3.5    5.3   5.4   160   252   160   254   121   281 0.54 - Competence protein  
G02171\_P             -            314 DsbD                 PF02683.10   211      0.44    8.8   2.9   1   1     0.001         1    7.5   2.9    11    58   187   233   164   244 0.85 - Cytochrome C biogenesis protein transmembrane region
